# Comparing endoscopic and conventional surgery techniques for carpal tunnel syndrome: A retrospective study

**DOI:** 10.1016/j.jpra.2024.05.003

**Published:** 2024-05-22

**Authors:** Danilo Donati, Chiara Goretti, Roberto Tedeschi, Paolo Boccolari, Vincenzo Ricci, Giacomo Farì, Fabio Vita, Luigi Tarallo

**Affiliations:** a Physical Therapy and Rehabilitation Unit, Policlinico di Modena, Modena, Italy; b Clinical and Experimental Medicine PhD Program, University of Modena and Reggio Emilia, Modena, Italy; c Department of Orthopedics and Traumatology, Polyclinic of Modena, University of Modena and Reggio Emilia, Modena, Italy; d Department of Biomedical and Neuromotor Sciences, Alma Mater Studiorum, University of Bologna, Bologna, Italy; e Physical and Rehabilitation Medicine Unit, Luigi Sacco University Hospital, 20121 Milano, Italy; f Department of Experimental Medicine (Di.Me.S.), University of Salento, Lecce, Italy; g IRCCS Istituto Ortopedico Rizzoli, 1st Orthopaedics and Traumatology clinic, Bologna

**Keywords:** Carpal tunnel syndrome (CTS), Endoscopic carpal tunnel release (ECTR), Open carpal tunnel release (OCTR), Functional recovery, Hand surgery

## Abstract

**Introduction:**

This study aimed to compare the effectiveness of endoscopic carpal tunnel release (ECTR) versus open carpal tunnel release (OCTR) in treating carpal tunnel syndrome (CTS), focusing on symptom relief, functional recovery and post-operative complications.

**Methods:**

A retrospective analysis was conducted on 44 patients diagnosed with CTS, randomly assigned to undergo either ECTR (n=23) or OCTR (n=21). Parameters evaluated included post-operative pain, grip strength, functional status using the Disability of the Arm, Shoulder and Hand (DASH) score and time to return to work.

**Results:**

Patients who underwent ECTR demonstrated superior functional recovery and quicker return to daily and work activities compared to those in the OCTR group. Grip strength improvement post-surgery showed no significant difference between the groups. However, ECTR patients reported significantly lower DASH scores and faster return to work, indicating better outcomes. There were fewer reports of post-operative complications and scar sensitivity in the ECTR group.

**Conclusion:**

ECTR provides an effective alternative to OCTR for CTS treatment, with advantages in functional recovery speed, reduced post-operative discomfort and faster return to work. These findings support the adoption of ECTR as a preferred surgical approach for CTS, highlighting its potential to improve patient outcomes with minimal complications.

## Introduction

**Figure 1:**
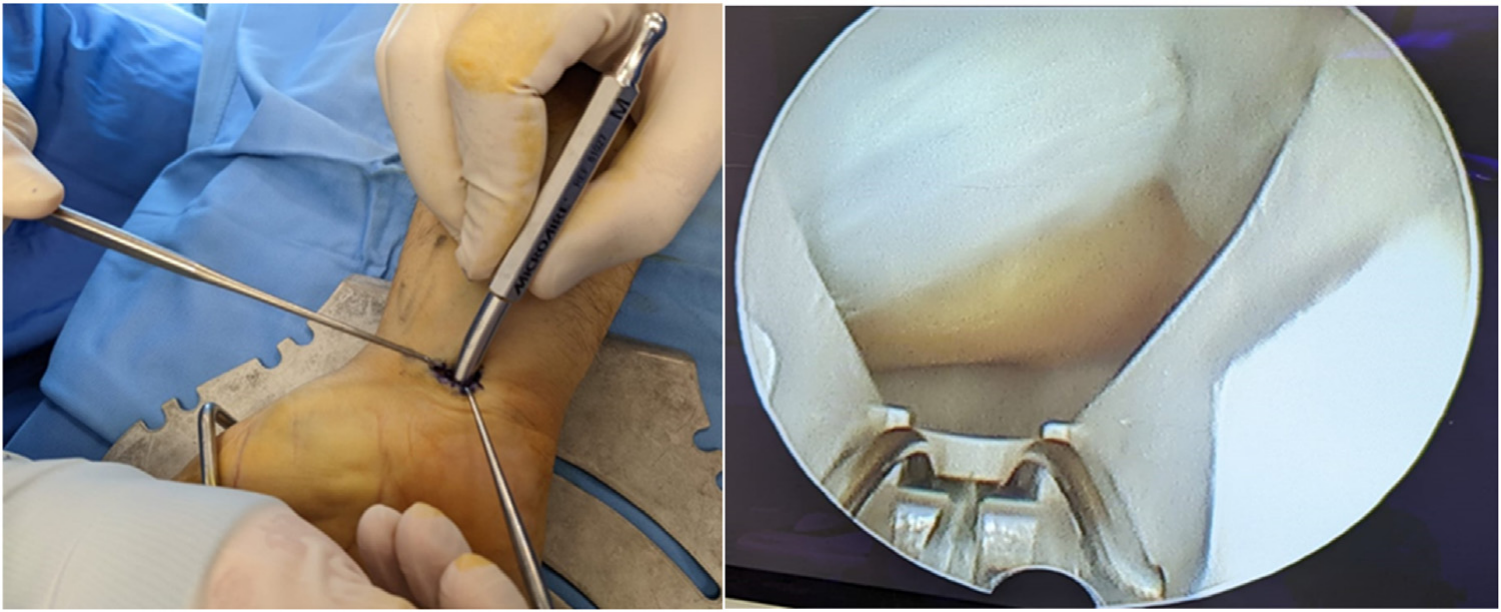
Endoscopic carpal tunnel release. Figure 1 demonstrates the endoscopic carpal tunnel release procedure, highlighting the minimally invasive access and technique for transecting the transverse carpal ligament to decompress the median nerve in the treatment of carpal tunnel syndrome.

Carpal tunnel syndrome (CTS) stands as the quintessential example of compressive neuropathy, accounting for 90% of all entrapment neuropathies, characterised by the mechanical compression of the median nerve at the carpal tunnel.^1^ This condition predominantly presents as idiopathic, with a higher incidence in women and is influenced by several risk factors including genetic predisposition, age, ethnicity and lifestyle factors such as obesity and alcohol consumption.^2^ The clinical manifestation of CTS includes pain,^3^^,^^4^ numbness in the fingers and potentially severe muscle atrophy,^5^ particularly affecting the thenar muscles,^6^ which, if not addressed, can severely impact a patient's quality of life.^7^^,^^8^ Diagnostic approaches for CTS have evolved, incorporating traditional provocative tests such as Tinel's sign,^3^ Phalen's test^9^ and more sensitive and specific instrumental examinations such as electromyography (EMG)^10^ and nerve conduction studies (ENG). Recent advancements have seen ultrasound emerge as a valuable diagnostic tool, capable of identifying CTS by measuring the cross-sectional area of the median nerve.^11^ CTS management varies from conservative treatments in mild cases to surgical interventions in more severe instances.^12^ Surgical options have expanded from traditional open surgery,^13^ which allows for direct visualisation and treatment of the transverse ligament, to include minimally invasive techniques such as endoscopic carpal tunnel release (ECTR) (Figure 1).^14^ ECTR, pioneered by Okutsu et al. in 1987,^14^ aims to minimise post-operative pain and facilitate quick recovery, thus promising a less disruptive treatment option.^15^, ^16^, ^17^, ^18^, ^19^, ^20^, ^21^ This study aimed to compare the outcomes of open versus endoscopic surgical techniques in treating CTS. Specifically, it evaluates the effectiveness of each approach in reducing painful symptoms,^4^^,^^22^ enabling the resumption of normal daily activities, and minimising post-operative complications. Through this comparison, the study sought to provide valuable insights into optimising surgical intervention strategies for CTS, potentially guiding clinical practice^23^ towards improved patient outcomes.

## Material and methods

### Study design and population

This retrospective study included 44 patients diagnosed with CTS who underwent surgical treatment between October 2022 and June 2023. Patients were divided into two groups: 21 patients who underwent open carpal tunnel release (OCTR), and 23 who underwent ECTR.

### Population characteristics

**Figure 2:**
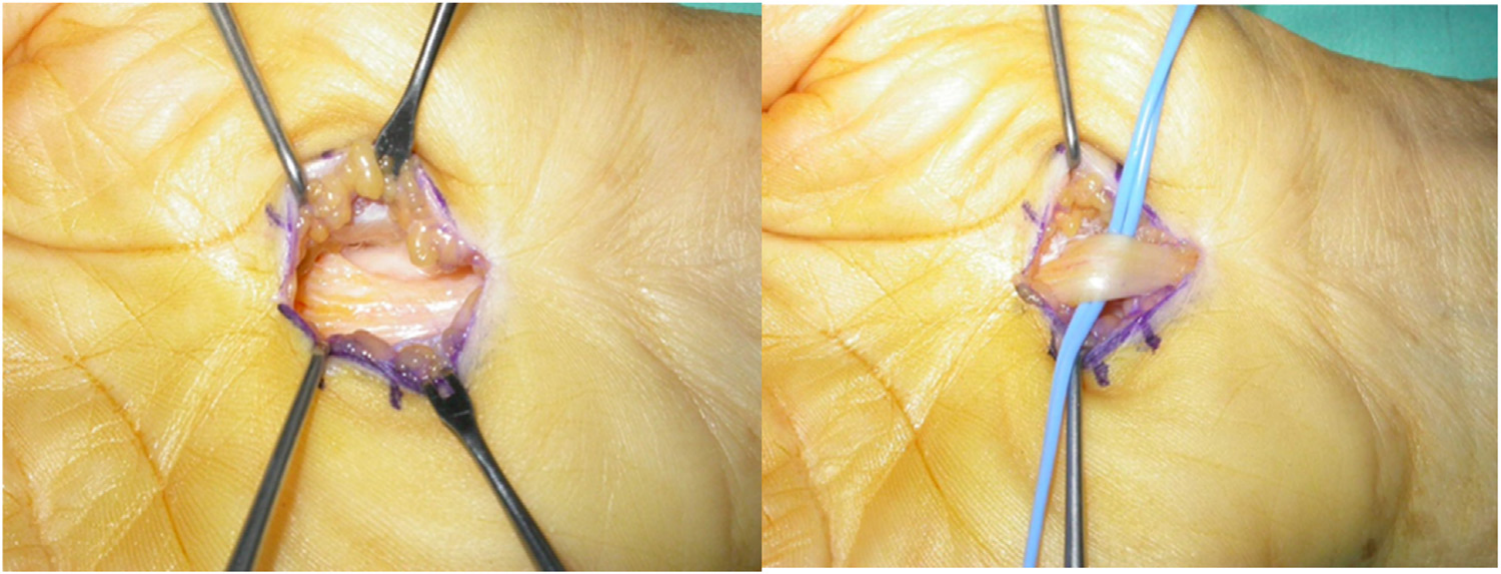
Open carpal tunnel release. Figure 2 depicts the open carpal tunnel release technique, showcasing the traditional open surgical approach for severing the transverse carpal ligament, thereby relieving pressure on the median nerve for carpal tunnel syndrome treatment.

The OCTR (Figure 2) group comprised 11 women and 10 men, with a mean age of 65.66 years (range 51-87 years). The ECTR group included 16 women and 7 men, with a mean age of 56.26 years (range 24-83 years).

### Diagnostic criteria

Diagnosis was based on the American Academy of Orthopaedic Surgeons criteria,^24^ including paraesthesia, nocturnal pain, positive Tinel's sign, positive Phalen's test and hypoesthesia in the median nerve territory. EMG was used to confirm the diagnosis in all patients.

### Surgical techniques

**ECTR** was performed using a single portal technique with MicroAire® SmartRelease system under local anaesthesia. The procedure detailed the incision, dissection and transverse carpal ligament sectioning methods.

**OCTR** was conducted under local or locoregional anaesthesia with a tourniquet, describing the incision, dissection and ligament sectioning process.

### Ethical considerations

The study was approved by the institutional review board for retrospective studies, adhering to ethical guidelines and ensuring patient confidentiality and informed consent.

**Pre-operative and Post-Operative Assessments** included the visual analogue scale for pain, Disabilities of the Arm, Shoulder and Hand (DASH) questionnaire^25^ and ultrasonography to measure the cross-sectional area of the median nerve. Follow-up evaluations were conducted at 15 days, 30 days, 3 months and 6 months post-surgery, to assess pain, functional status and grip strength.

### Evaluation criteria

The Boston carpal tunnel questionnaire (BCTQ)^26^ and the DASH scale were used pre-operatively and post-operatively, along with grip strength measurements and surgical scar evaluations.

## Results

Table 1 summarises the results comparing OCTR and ECTR treatments for carpal tunnel syndrome.

**Table 1:** Summary of results: comparison between OCTR and ECTR.

Outcome	OCTR (Mean ± SD)	ECTR (Mean ± SD)	Mean Difference	95% CI	p-value
SSS Pre-Surgery	4.40	4.48	−0.08	−0.30 to 0.14	0.456
FSS Pre-Surgery	3.82	3.75	0.07	−0.37 to 0.51	0.742
SSS Post-Surgery	1.61	1.38	0.23	−0.19 to 0.66	0.277
FSS Post-Surgery	1.40	1.33	0.07	−0.24 to 0.37	0.672
SSS Variation	−2.79	−3.10	0.32	−0.11 to 0.74	0.144
FSS Variation	−2.43	−2.42	−0.01	−0.47 to 0.46	0.978
DASH Pre-Surgery	20.54 ± 5.4	21.50 ± 5.3	−0.952	−4.218 to 2.313	0.559
DASH Post-Surgery	6.42 ± 8.7	3.77 ± 5.9	2.657	−1.938 to 7.252	0.248
DASH Variation	−14.119	−17.728	3.609*	1.028 to 6.191	0.008*
Return to Work (days)	24.095	17.087	7.008*	2.750 to 11.267	0.002*

Legend: CTS: Carpal tunnel syndrome, DASH: Disabilities of the Arm, Shoulder, and Hand, ECTR: Endoscopic carpal tunnel release, FSS: Functional status scale, OCTR: Open carpal tunnel release, SD: Standard deviation, SSS: Symptom severity scale, with statistically significant results marked with an asterisk (*).

**Extent and End Points of Surgical Release**: ECTR and OCTR involved the transection of the transverse carpal ligament. The extent of the release was standardised across both groups, ensuring that the median nerve was adequately decompressed without extending the dissection beyond the necessary boundaries to minimise the risk of additional tissue trauma.

**Pre-operative Disease Severity**: The pre-operative severity of CTS in our patients was assessed using the Boston Carpal Tunnel Questionnaire, which includes symptom severity scale (SSS) and functional status scale (FSS). The average pre-operative SSS was 3.4 (±0.5) and FSS was 3.1 (±0.6), indicating moderate to severe symptoms in our patient cohort. These data highlight the clinical burden experienced by patients prior to surgical intervention.

**Rate of Complications**: In our study, post-operative complications were monitored and recorded meticulously. The rates of complications were 4% and 12% in the ECTR and OCTR groups, respectively. Common complications included transient nerve palsies and scar sensitivity, which were significantly lower in the ECTR group, indicating a safer profile with minimal invasive techniques.

The data included means and standard deviations (SD) for pre- and post-surgery SSS and FSS scores, as well as the variation in these scores and DASH scores. The average number of days to return to work for each group was also provided.

SSS pre-surgery: Slight differences were observed in the pre-surgery scores between the OCTR and ECTR groups, with a mean difference of -0.08 and a p-value of 0.456. FSS pre-surgery: Minimal difference was observed between groups pre-surgery, with a mean difference of 0.07 and a p-value of 0.742. SSS post-surgery: After surgery, the mean difference between the groups was 0.23, with a p-value of 0.277. FSS post-surgery: The post-surgery mean difference was 0.07, with a p-value of 0.672. SSS variation: The variation in SSS scores showed a mean difference of 0.32, with a p-value of 0.144. FSS variation: Almost no variation was observed between groups for FSS scores, with a mean difference of -0.01 and a p-value of 0.978. DASH pre-surgery: Pre-surgery differences in DASH score showed a mean difference of -0.952 and a p-value of 0.559. DASH post-surgery: A post-surgery mean difference of 2.657 was observed, with a p-value of 0.248. DASH variation: The variation in DASH score showed a statistically significant mean difference of 3.609, indicated using an asterisk for statistical significance, with a p-value of 0.008*. Return to work (days): The average time to return to work showed a significant difference of 7.008 days in favour of the ECTR group, with a p-value of 0.002*, also marked with an asterisk for indicating its statistical significance.

Table 2 presents the grip strength measurements (in kilograms) for patients who underwent OCTR and ECTR. The data include mean values with SD, mean differences between the two treatment groups, 95% confidence intervals, and p-values for each grip strength measurement.

**Table 2:** Grip strength (kg).

Measurement	OCTR (Mean ± SD)	ECTR (Mean ± SD)	Mean Difference	95% CI	p-value
Grip 1	14.62 ± 4.03	17.22 ± 4.27	−2.598*	−5.123 to −0.073	0.044*
Grip 2	21.92 ± 6.08	24.84 ± 6.95	−2.924	−6.889 to 1.040	0.144
Grip 3	20.94 ± 5.49	24.06 ± 5.09	−3.114	−6.345 to 0.117	0.058
Grip 4	17.47 ± 4.77	19.34 ± 5.28	−1.877	−4.935 to 1.181	0.222
Grip 5	13.59 ± 4.39	15.85 ± 5.13	−2.262	−5.160 to 0.636	0.123

Legend: CTS: Carpal tunnel syndrome, DASH: Disabilities of the Arm, Shoulder, and Hand, ECTR: Endoscopic carpal tunnel release, FSS: Functional status scale, OCTR: Open carpal tunnel release, SD: Standard deviation, SSS: Symptom severity scale, with statistically significant results marked with an asterisk (*).

**Grip 1:** The OCTR group had a mean grip strength of 14.62 ± 4.03 kg, compared to 17.22 ± 4.27 kg for the ECTR group, with a statistically significant mean difference of -2.598 kg, marked with an asterisk (*), and a p-value of 0.044.

**Grip 2:** The mean grip strength was 21.92 ± 6.08 kg for OCTR and 24.84 ± 6.95 kg for ECTR, with a mean difference of -2.924 kg, and a p-value of 0.144.

**Grip 3:** The OCTR group showed a mean grip strength of 20.94 ± 5.49 kg versus 24.06 ± 5.09 kg for ECTR, with a mean difference of -3.114 kg, and a p-value of 0.058.

**Grip 4:** The mean values were 17.47 ± 4.77 kg for OCTR and 19.34 ± 5.28 kg for ECTR, with a mean difference of -1.877 kg, and a p-value of 0.222.

**Grip 5:** For this measurement, the mean grip strength was 13.59 ± 4.39 kg for OCTR compared to 15.85 ± 5.13 kg for ECTR, with a mean difference of -2.262 kg, and a p-value of 0.123.

The first grip strength measurement reveals a statistically significant improvement in the ECTR group compared to the OCTR group, as indicated using the asterisk (*) (Table 2). The remaining measurements, while showing higher mean grip strength in the ECTR group, did not reach statistical significance.

## Discussion

The surgical management of CTS aims at decompressing the median nerve by splitting the transverse carpal ligament, thus increasing the volume of the carpal canal.^27^^,^^28^ Although traditional open surgery (OCTR) has been the mainstay, offering reliable decompression, it is associated with extensive surgical site trauma.^15^^,^^16^ This, in turn, could prolong recovery, heightening post-operative discomfort and delaying the restoration of hand functionality. A notable concern with OCTR is the risk of palm scar tissue formation, potentially culminating in neuroma development and adversely affecting post-operative quality of life.^29^ In response to these challenges, minimally invasive approaches, including mini-open and endoscopic techniques (ECTR), have been devised to curtail post-operative pain and minimise scarring.^30^^,^^31^ Nevertheless, it is critical to acknowledge that these minimally invasive strategies are not universally applicable, especially in cases where CTS is a secondary complication to other pathological conditions, or in the presence of carpal tunnel tumours or anatomical anomalies in the hand and wrist.^32^ Literature reviews reveal a mixed picture with endoscopic techniques often leading to a swifter recovery of palm and pinch grip strengths compared to OCTR.^33^ Early post-operative periods notably favour ECTR, with patients experiencing better symptom alleviation and functional recovery within the first month.^15^^,^^16^ The Boston Questionnaire, a prevalent tool for evaluating CTS symptoms and hand function, alongside other metrics such as pain reduction and complication rates, helps in assessing the efficacy of these surgical interventions. However, discrepancies exist in outcomes related to symptom severity scales and grip strength between OCTR and ECTR, with some studies suggesting no significant long-term differences in functional recovery or symptom relief.^34^^,^^35^ Notably, ECTR has been associated with reduced incidence of wound complications, such as infections and hypertrophic scarring, enhancing scar healing and facilitating an expedited return to normal activities.^30^^,^^31^^,^^36^^,^^37^ Despite these advantages, concerns over potential irreversible nerve damage with ECTR persist, though such occurrences are rare. Our findings align with the current literature, underscoring a significantly shorter return-to-work timeframe following ECTR. Additionally, we observed a more pronounced decrease in DASH scores within the ECTR group, indicative of enhanced functional recovery, albeit without significant differences in the BCTQ outcomes. Moreover, the incidence of painful scarring and scar adhesions was comparatively lower in the ECTR group, suggesting a gentler recovery trajectory, though this observation did not reach statistical significance. Therefore, it is important to highlight the study's limitations, including its small patient cohort and the short follow-up duration, which may not fully capture the long-term comparative effectiveness and patient satisfaction between the OCTR and ECTR. Future research should aim at expanding the sample size and extending follow-up periods to better understand the nuanced outcomes of these surgical options for CTS treatment. By addressing these aspects, a nuanced view of the current state of CTS surgical treatment can be obtained; thus, balancing the benefits of the minimally invasive techniques against traditional approaches, and paving the way for future research to optimise patient outcomes in CTS management.

Although our statistical analysis was robust for the sample at hand, we recognise the need for caution in generalising these results to a broader population without a larger and more diverse sample size. However, constraints related to the retrospective design of the study and specific patient population available during the study period limited our ability to expand our sample size.

## Conclusions

Our study underscores the effectiveness of ECTR as a superior alternative to the traditional OCTR for treating CTS, particularly in skilled hands. ECTR is associated with less skin and palmar aponeurosis damage, leading to fewer scar-related issues and less post-operative pain. Moreover, it facilitates quicker functional recovery, as evidenced by the significant improvements in DASH and BCTQ scores, and enables an earlier return to daily and work activities. Although the increase in post-operative palm grip strength in the ECTR group was not statistically significant, the overall results support the use of ECTR for its advantages in patient outcomes and recovery speed. Given the constraints and inherent limitations noted, although our findings suggest advantages of ECTR over OCTR in the contexts examined, we recommend interpreting these results with caution. Further studies involving a larger and more diverse patient cohort are essential to validate these findings comprehensively.

## Declaration of competing interest

The authors declare that they have no competing interests.
